# A novel method for the remote measurement of trismus in clinical trials using mobile phone cameras

**DOI:** 10.1007/s00784-023-05272-z

**Published:** 2023-11-10

**Authors:** Xiang Li, Caroline Kristunas, Gabriel Landini, Annika Kroeger, Thomas Dietrich

**Affiliations:** 1 School of Dentistry, Institute of Clinical Sciences, College of Medical and Dental Sciences, University of Birmingham, Birmingham, UK; 2 Institute of Cancer and Genomic Studies, University of Birmingham, Birmingham, UK; 3 Department of Oral Surgery, Birmingham Dental Hospital, Birmingham Community Healthcare NHS Foundation Trust, Birmingham, UK

**Keywords:** Trismus, Measurement, Reliability, Validity, Third molar

## Abstract

**Objective:**

To evaluate the reliability and validity of a novel method for remotely measuring trismus.

**Materials and methods:**

We recruited 60 volunteers who took three types of photographs at a fixed restricted jaw position mimicking limited mouth opening, including one selfie and one portrait with or without a reference frame. Additionally, the interincisal distance and the width of the upper central incisors were measured with a ruler, as per common practice. Measurements of trismus were made using image analysis software comparing different types of photos and calibration methods. Intraclass correlation coefficient (ICC) and 95% limits of agreement (LoA) with 95% confidence interval were calculated to evaluate reliability and validity.

**Results:**

The proposed method demonstrated high reliability (ICC 0.998; 95% CI 0.997, 0.999). Calibration of photographs using at least a baseline photograph with an external reference frame yielded unbiased measurements and minimised variability. The use of selfies compared to portrait photos also increased variability.

**Conclusion:**

The measurement of trismus can be performed using images taken remotely by patients using their mobile phone cameras. The proposed method is highly accurate, with best results obtained by using a reference frame for calibration of portrait photographs.

**Clinical relevance:**

We propose an easy, cheap, and accurate method that allows for remote and frequent monitoring of trismus in clinical studies using patients’ mobile phones.

## Introduction

The surgical removal of lower third molars (LM3) is one of the most frequently performed clinical procedures in the field of oral surgery. The procedure is associated with significant postoperative morbidity, including pain, trismus, swelling, and a risk of surgical site infection/alveolar osteitis, all of which have an adverse impact on the postoperative quality of life of patients. Much clinical research is therefore focussed on the evaluation of interventions that are aimed at minimising these untoward sequelae of LM3 surgery. Clinical trials in this area typically employ a variety of outcome measures to assess postoperative morbidity.

Trismus, i.e., the reduction in mouth opening during the postoperative recovery period, is a key outcome measured in most of these trials. Severely restricted mouth opening has a negative impact on daily activities and is therefore an important domain of the postoperative quality of life of patients [1]. Furthermore, its clinical measurement is straightforward, and one could argue that it has the advantage of being a continuous measure that is less subjective or fuzzy than other outcome measures such as pain or alveolar osteitis [2].

However, the measurement of trismus by an investigator requires study participants to attend the clinic, which, if not part of routine care, places an additional burden on participants, with implications for study resources, patient compliance, and completeness of data. In practical terms, this also limits the frequency of assessments during the postoperative period and therefore, the accuracy with which the postoperative recovery can be monitored. The widespread adoption of smartphones with built-in cameras may afford an opportunity for remote and frequent monitoring of mouth opening during the postoperative healing following LM3 surgery and other clinical applications.

Therefore, we hypothesised that mouth opening could be monitored remotely by patients with high accuracy using photographs taken with mobile phones. We propose here a method to remotely assess trismus in clinical research studies. The purpose of this study was to evaluate its reliability and validity as a function of some of its parameters.

## Methods

The measurement of mouth opening involves the measurement of the interincisal distance. If the incisal edges of both the upper and lower incisors are visible on a photograph, the linear measurement of the interincisal distance is straightforward with standard image analysis software. The challenge is to calibrate these measurements in order to obtain absolute measures of mouth opening (in mm) or assess changes in mouth opening over time.

**Fig. 1:**
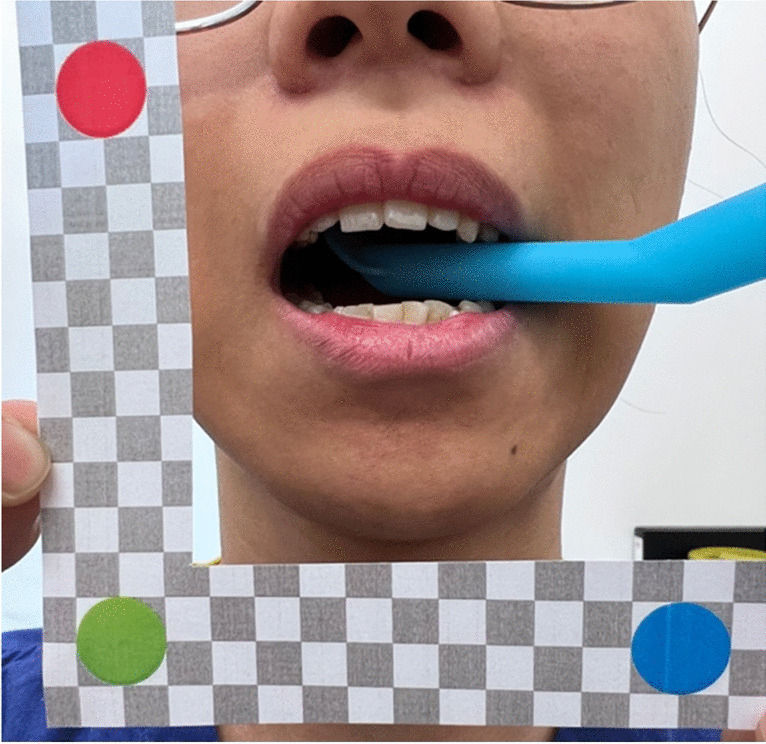
An example of portrait taken with the reference frame

The method we propose here involves the use of an external reference frame and the incisor width as a fixed anatomical landmark for calibration. The reference frame (Fig. 1) features a grey and white grid of known dimensions. The addition of three landmarks (blue, red, and green discs) allows for automatic positioning and calibration using ImageJ [3], which is available on request from the authors. In a clinical research application, a photograph of the patient’s mouth opening would be taken by an investigator with the reference frame, so the landmarks can be used for calibration of the image. Additionally, the incisor width can be determined from the calibrated image to calibrate subsequent images taken by the patient remotely (without the reference frame). In the present study, we evaluated the reliability of this method and compared the validity of variations of the method, including using clinical measurements of the incisor width as a reference and using portrait vs ‘selfie’ photographs.

### Study design and procedures

This validation study was approved by the Science, Technology, Engineering and Mathematics Ethical Review Committee at the University of Birmingham (AER number: ERN_22-0198). Volunteers were recruited from staff and students from Birmingham Dental Hospital/School of Dentistry, University of Birmingham, between April and June in 2022. All participants gave written informed consent for study participation.

Volunteers were asked to hold a reproducible jaw position by gently biting on either end of a standard suction tube and holding it using their molar teeth, resulting in two fixed jaw positions. A series of photographs in each of the two jaw positions were then taken, including (i) a photograph with the reference frame taken by an investigator (hereafter called ‘portrait with frame’, Fig. 1), (ii) a photograph without the reference frame taken by an investigator (hereafter called ‘portrait without frame’), and (iii) a selfie without the reference frame taken by the participant. All photographs were taken using the respective participant’s mobile phone. In addition, an investigator measured the width of both central incisors and the mouth opening (distance between the incisal edges of the upper left central incisor and the lower left central incisor) to the nearest millimetre using a standard ruler. Volunteers were given a QR code to upload all photographs onto a database system (REDCap). Study data were collected and managed using REDCap electronic data capture tools hosted at the University of Birmingham [4, 5].

### Data extraction from photographs

Photographs were processed via ImageJ software [3] (version 1.53s on the macOS platform), and the mouth opening (the distance between the incisal edges of the upper and lower left central incisors) as well as the incisal widths (the horizontal distance between the distal edges of the upper central incisors) was measured. Readings were rounded to the nearest hundredths and presented in millimetres. Measurements on images taken with the reference frame (portrait with frame) were automatically calibrated using a macro (available from the authors on request). Both the portraits without frame and the selfies were calibrated using the widths of the upper central incisors as the reference. This was done in two different ways using either the clinical measurement (ruler calibration) or the measurement taken from the macro-calibrated portrait with frame (macro-calibration). Thus, there were 5 different measurements of the mouth opening for each jaw position: (i) the gold-standard ‘direct’ measurement taken from the calibrated portrait with frame (M1), (ii) the measurement taken from the portrait without frame using the macro-calibrated incisal width as a reference (M2), (iii) the measurement taken from the portrait without frame using the ruler-calibrated incisal width as a reference (M3), (iv) the measurement taken from the selfie using the macro-calibrated incisal width as a reference (M4), and (v) the measurement taken from the selfie using the ruler-calibrated incisal width as a reference (M5).

These measurements correspond to different implementation scenarios in terms of who would take what photographs in a clinical research application (Table 1). M1 is the gold standard where all photographs are taken by a third person (e.g., clinician in clinic and helper at home) with a reference frame, allowing direct macro-calibration of each photograph. M2 is a scenario where an initial portrait photograph with a reference frame is taken on site by a clinician/investigator, which is then used to calibrate subsequent portrait photos taken without the frame using the incisor width as a reference. M3 is where only a clinical measurement of the incisal width is taken on site and used for subsequent calibration. M4 and M5 use measurements taken from selfies rather than portraits, using the same calibration methods as M2 and M3, respectively.

**Table 1:** Summary of measurement methods corresponding to different clinical scenarios of measuring mouth opening

Measurement type	Type of photo at clinic	Type of photo taken remotely	Calibration type
M1 (gold standard)	Portrait w/ frame	Portrait w/ frame	Macro
M2 (portrait–macro-calibration)	Portrait w/ frame	Portrait w/o frame	Macro
M3 (portrait–ruler calibration)	None	Portrait w/o frame	Ruler
M4 (selfie–macro-calibration)	Portrait w/ frame	Selfie w/o frame	Macro
M5 (selfie–ruler calibration)	None	Selfie w/o frame	Ruler
M6 (manual frame calibration)	Portrait w/ frame	Portrait w/ frame	Manually using frame

Because the automatic macro-calibration may fail in a small number of images due to background noise or non-homogeneous background or the reference frame not being fully captured in the image, we also assessed the validity of a manual calibration using the grey and white grid of the frame as the reference (M6), compared to the gold standard frame calibration.

### Statistical methods and data analysis

#### Sample size calculation

The reliability of the gold standard (M1) was assessed using the intraclass correlation coefficient (ICC). The reliability was expected to be high, 0.9; therefore, to estimate the ICC and 95% confidence interval with a precision of 0.05, a random sample of 42 images with the reference frame was needed, with each of them measured three times [6]. As two photographs using M1 would be collected from each participant, a minimum of 21 participants was required.

To assess the agreement between each method and the gold standard, the limits of agreement method for multiple observations per individual, where the true value varies, proposed by Bland and Altman [7] were used. Two measurements were taken per participant (at different jaw positions). Since the number of measurements per participant was small in comparison to the number of participants, the limits of agreement should not differ considerably from those calculated assuming independent observations [7]. Therefore, we aimed to obtain a total of 100 observations, as recommended by Bland [8], to estimate the limits of agreement with a 95% confidence interval of approximately ± 0.34*(standard deviation of the mean differences between measurements by different methods). A total of 50 participants were therefore required.

Since the reliability and the agreement were both investigated, a minimum of 50 participants was therefore required.

#### Statistical analysis

STATA 17 was used to conduct the analysis. For the reliability of M1, the ICC was estimated from a two-way random effects model. The mean differences (gold standard minus comparator method) and their standard deviations were calculated. To estimate the limits of agreement between the methods, the method described by Bland and Altman for multiple observations per individual was used [7]. A sensitivity analysis was conducted, assuming independent observations, as was assumed in the sample size estimation.

## Results

We recruited 60 volunteers who uploaded photographs to the database. The number of photographs available for analysis for each modality varied due to issues such as some photographs being excluded owing to upload errors, background noise in images prohibiting macro-calibration, reference frame not being completely in the picture, or upper or lower incisors not captured sufficiently.

### The reliability of the gold standard (M1) measurement

The reliability of M1 was estimated using 42 images from 21 participants. The result showed an extremely high reliability with an ICC of 0.998 (95% CI 0.997, 0.999).

### Agreement of the conventional ruler measurement of incisal width with gold standard (M1)

**Fig. 2:**
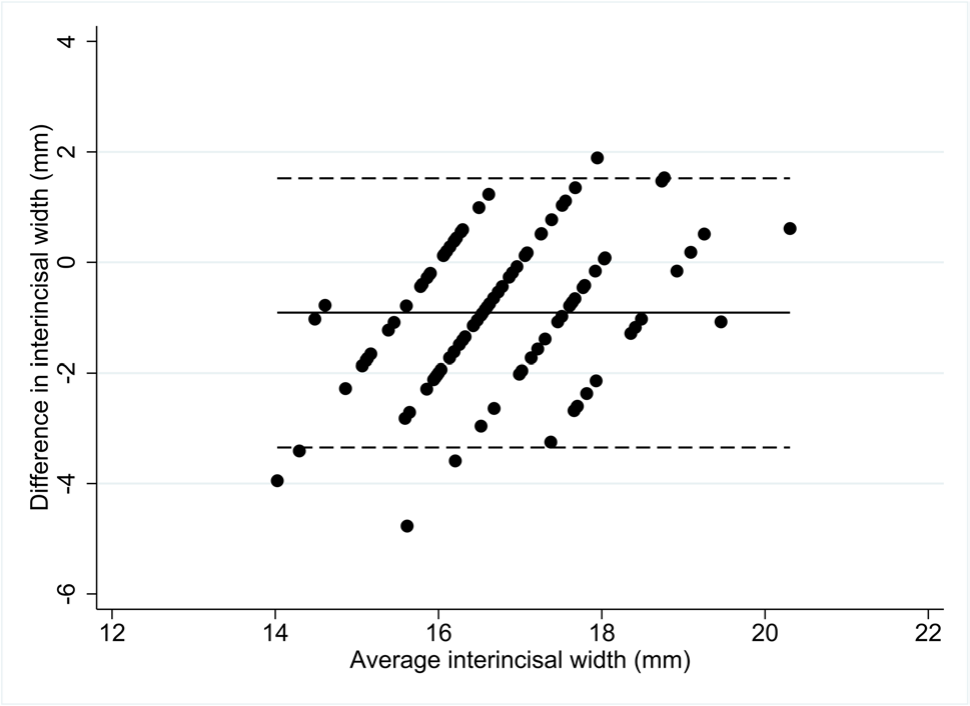
Bland-Altman plot comparing the incisal width measured by the conventional ruler measurement compared to the gold standard (M1). The mean difference and the limits of agreement were presented on the *Y*-axis. The conventional ruler measurement overestimated the incisal width by 0.91 mm (as the solid line in the middle shown) with limits of agreement spanning 4.87 mm (as the lower and upper dotted line demarcated)

The mean difference between the gold standard measurement (M1) and the conventional ruler measurement of the incisal width was − 0.91 mm (95% CI − 1.16 mm, − 0.66 mm). The lower and upper limits of agreement were − 3.35 mm (95% CI − 3.77 mm, − 2.93 mm) and 1.52 mm (95% CI 1.10 mm, 1.94 mm) respectively (Fig. 2).

### Agreement with the gold standard (M1) measurement of mouth opening

**Fig. 3:**
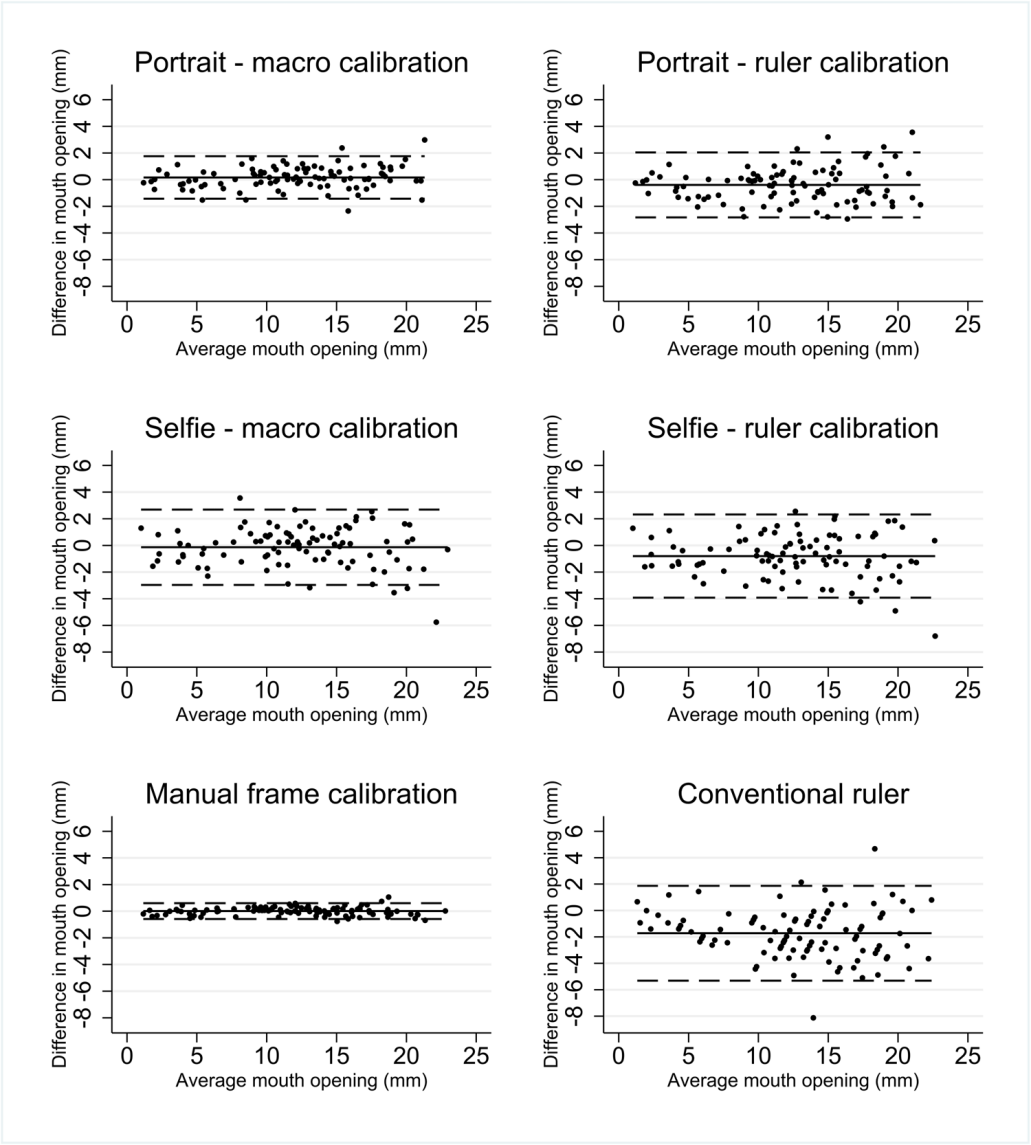
Bland-Altman plots comparing the gold standard (M1) to various calibration/photograph types. Compared with the gold standard (M1), manual frame calibration (M6) performed best with the least bias and the narrowest limits of agreement of all of the comparator methods

The mean difference and limits of agreement between the methods are provided in Table 2 and shown in Fig. 3. Method M6, the manual calibration using the reference frame, performed well, with the least bias and the narrowest limits of agreement of all of the methods. Both the portrait and selfie photographs using the macro-calibration (M2 and M4) had little bias, but M2 provided narrower limits of agreement. M3 had similar limits of agreement, but on average gave slightly larger estimates of the mouth opening than the gold standard. M5 overestimated the mouth opening by 0.8 mm on average and had wider limits of agreement than the other calibrated methods. The conventional ruler measurement performed the poorest, overestimating the mouth opening by 1.7 mm and with limits of agreement spanning 7.19 mm.

**Table 2:** Mean difference (95% confidence interval) and limits of agreement (95% confidence interval) between the gold standard (M1) and alternative methods for the measurement of mouth opening

Comparisons between comparator methods	Mean difference (mm) (95% CI)	Adjusted 95% limits of agreement (mm) (95% CI)	Number (*n*) of valid images
M1 vs M2	0.16 (**−**0.00, 0.33)	−1.43 (**−**1.71, **−**1.15), 1.76 (1.48, 2.04)	98
M1 vs M3	−0.39 (**−**0.65, **−**0.14)	−2.83 (**−**3.26, **−**2.40), 2.05 (1.62, 2.48)	98
M1 vs M4	−0.14 (**−**0.44, 0.17)	−2.97 (**−**3.48, **−**2.46), 2.69 (2.18, 3.20)	95
M1 vs M5	−0.80 (**−**1.14, **−**0.46)	−3.92 (**−**4.49, **−** 3.35), 2.33 (1.76, 2.90)	94
M1 vs M6	0.01 (**−**0.05, 0.07)	−0.58 (**−**0.68, **−**0.48), 0.60 (0.50, 0.70)	100
M1 vs convention ruler measurement	−1.72 (**−**2.09, **−**1.36)	−5.32 (**−**5.94, **−**4.70), 1.87 (1.25, 2.49)	100

### Sensitivity analysis

Ignoring the multiple images taken from the same participant in the agreement analysis did not result in a change to the conclusions.

## Discussion

In this study, we demonstrate excellent reliability and validity of a photographic method of ascertaining measurements of trismus (mouth opening) remotely with the use of patients’ mobile phone cameras. The method requires minimal resources and is very easy to implement. The use of a reference frame allows for the most accurate calibration of images taken.

Our results suggest that perhaps the most practical method for remote assessment of trismus in a clinical research application would be the use of a photograph taken with a reference frame by an investigator, which allows the use of a fixed anatomical landmark (incisor width) for calibration of subsequent images taken by patients remotely. Care should be taken to take this initial photograph against a white background, in order to avoid background noise. This is because the background noise, which in this context refers to any undesired environment features, might confuse the calibration of the image in the detection of the landmarks (coloured discs mentioned above), thus interfering with macro-calibration. This approach obviates the need for supplying each patient with a reference frame for remote use and simplifies the procedures for patients, while providing unbiased results with minimal additional variability. Relying on clinical ruler measurements of incisor width for image calibration introduces additional variability and should only be used if the use of a reference frame is deemed impractical. While the use of selfie photographs yielded satisfactory results, portrait photos taken by a third person can minimise variability, probably due to better framing of photographs and better-quality cameras.

Trismus is an important clinical endpoint in clinical trials of the morbidity associated with third molar surgery. Traditionally, its measurement requires patients to attend the clinic for follow-up appointments, which has resource implications and typically results in measurements only being available for one or two postoperative days. The remote assessment of trismus allows for more frequent measurements and therefore more accurate monitoring of the onset and resolution of trismus during the postoperative healing period. We had previously reported satisfactory accuracy of remote measurements taken with a simple cardboard scale [9]. However, the ubiquitous availability of mobile phones with high quality cameras obviates the need for any additional equipment to be managed by the patient, which should increase the acceptability and reduce missing data in an application.

One important limitation of the proposed method is the fact that a small minority of participants do not normally expose the incisal edges of both upper and lower incisors at full mouth opening. We found that asking these volunteers to expose the incisal edges by moving their lips tended to result in jaw movement leading to slightly smaller mouth openings. In a clinical trial application, we would therefore recommend restricting the study to patients who normally expose upper and lower incisors to allow the measurements described here. Additionally, due to the upload errors and background noise in the images, a small number of images were unsuitable for data extraction and therefore excluded from further analysis.

This resulted in the number of samples for four of the comparisons to fall below 100 as mentioned above, which led to slightly wider confidence intervals around the limits of agreement than were planned. However, this does not affect the interpretation of the findings reported here.

## Conclusion

In this study, we propose a method for the remote measurement of trismus using patients’ mobile phone cameras and readily available image analysis software. The method has excellent reliability and validity. The accuracy is highest with the use of a reference frame for calibration and portrait rather than selfie photos.

## Data Availability

Data are available from the authors upon reasonable request.
